# FoxM1 promotes Wnt/β‐catenin pathway activation and renal fibrosis via transcriptionally regulating multi‐Wnts expressions

**DOI:** 10.1111/jcmm.15948

**Published:** 2021-01-12

**Authors:** Hongyan Xie, Naijun Miao, Dan Xu, Zhuanli Zhou, Jiayun Ni, Fan Yin, Yanzhe Wang, Qian Cheng, Panpan Chen, Jingyao Li, Peiqing Zheng, Li Zhou, Jun Liu, Wei Zhang, Xiaoxia Wang, Limin Lu

**Affiliations:** ^1^ Department of Physiology and Pathophysiology School of Basic Medical Sciences Fudan University Shanghai 200032 China; ^2^ Department of Nephrology Shanghai Tong Ren Hospital Shanghai Jiao Tong University School of Medicine Shanghai 200336 China; ^3^ Shanghai Kidney Development and Pediatric Kidney Disease Research Center Shanghai 201102 China

**Keywords:** FoxM1, renal fibrosis, Wnts, β‐catenin

## Abstract

The activation of Wnt/β‐catenin pathway plays a pivotal role in promoting renal fibrosis. The activation of Wnt/β‐catenin pathway relies on the binding of Wnts to Frizzled receptors on cell membrane. However, the factor regulating Wnts production remains unclear. Here, we demonstrated that transcriptional factor FoxM1 was significantly increased in obstructed kidneys and patients' kidneys with fibrosis. The up‐regulation of FoxM1 mainly distributed in tubular epithelial cells. Pharmacological inhibition of FoxM1 down‐regulated multi‐Wnts elevation in UUO mice and attenuated renal fibrosis. In cultured renal tubular epithelial cells, overexpression of FoxM1 promoted 8 Wnts expression, while knock‐down on FoxM1‐suppressed multi‐Wnts including Wnt1, Wnt2b and Wnt3 expression induced by Ang II. Chromatin immunoprecipitation PCR confirmed that FoxM1 bound to Wnt1, Wnt2b, Wnt3 promoters and luciferase assay further identified that the transcriptions of Wnt1, Wnt2b and Wnt3 were regulated by FoxM1. Thus, our findings show that multi‐Wnt family members were regulated by transcriptional factor FoxM1. FoxM1 might be a key switch for activating β‐catenin pathway and renal fibrosis. Therefore, FoxM1 might be a potential therapeutic target in manipulating renal fibrosis.

## 1 INTRODUCTION

Chronic kidney disease (CKD) is currently a worldwide health concern due to affecting approximately 10% of the global population.^1^ Renal fibrosis is the common feature of CKD and the final consequence of excessive extracellular matrix (ECM) production and accumulation in interstitial spaces of kidney. Multi factors, including the activation of Wnt/β‐catenin signalling pathway, intrarenal renin‐angiotensin system, infiltration of myeloid inflammatory cells, sustained inflammation in local tissue and activation of interstitial fibroblasts, have been reported to contribute to the progression of renal fibrosis.^2^, ^3^, ^4^

It has been recently well documented that the activation of Wnt/β‐catenin signalling pathway is implicated in renal fibrosis.^5^, ^6^ Once activated, β‐catenin translocates into nucleus and acts as a transcriptional co‐activator to enhance multiple fibrotic gene expressions. The activation of Wnt/β‐catenin signalling pathway relies on the bindings of Wnts to Frizzled receptors on cell membrane. Wnts are secretory glycoproteins, and currently, 19 members have been identified in Wnt families.^6^, ^7^, ^8^, ^9^ Previous studies have showed that multi‐Wnt members are elevated in the pathological condition of CKDs.^10^, ^11^, ^12^ However, the factor lined on the upstream which regulates Wnts production remains unclear.

Forkhead Box M1 (FoxM1), a member of the Forkhead family, is a representative proliferation‐related transcription factor.^13^ FoxM1 is known as an important factor in regulating cell cycle transition and chromosome segregation.^14^, ^15^, ^16^ FoxM1 has been found to be expressed in alveolar epithelial cells.^17^ Recent study indicated that FoxM1 provoked injured proximal tubular proliferative repair in acute kidney injury (AKI).^18^ Bioinformatic analysis results showed that there are computative binding sites of FoxM1 on all human Wnts promoter regions and 13 of 19 rat Wnts promoter regions. In unilateral ureteral obstruction (UUO)‐induced renal fibrosis mice, our preliminary study result showed that FoxM1 was persistently increased in the fibrotic kidneys.

Based on the clue, the current study clarified that FoxM1 was an essential regulator of Wnts. Persistent increase of FoxM1 up‐regulated the expression of multi‐members of Wnts. Increased Wnts, as a consequence, activated β‐catenin signalling pathway in renal tubular epithelial cells and then promoted renal fibrogenesis. The up‐regulation of FoxM1 was also observed in biopsy samples with renal fibrosis.

## 2 MATERIALS AND METHODS

### 2.1 Animal models

Male C57BL/6J mice (8‐10 weeks old, 20‐25 g) were purchased from Animal Department of Fudan University (Shanghai, China). UUO mice were established as previously described. After anesthetized, the left ureter was exposed and ligated. The sham group was operated in the same procedure, except the left ureter ligation. Mice were administered with either DMSO or FoxM1 inhibitor Siomycin A via the i.p. injection at a dose of 6.25 mg/kg bodyweight at designated time points (days 1, 5, 9, 13) after UUO surgery. At 14 days, the mice were euthanized for the renal tissue harvest. Folic acid (FA) mice received a single intraperitoneal injection of FA (250 mg/kg bodyweight) dissolved in a 0.3 M sodium bicarbonate solution and were euthanized at 28 days. All animal experiments were conducted following to the Guidance for Care and Use of Laboratory Animals of Fudan University. The protocols complied with the Criteria of the Medical Laboratory Animal Administrative Committee of Shanghai and were approved by the Ethics Committee for Experimental Research of Shanghai Medical College, Fudan University.

### 2.2 Cell culture

NRK‐52E, a rat renal tubular epithelial cell line, was obtained from the Institute of Biochemistry and Cell Biology (Shanghai, China). The cells were cultured in DMEM media supplemented with 10% FBS at 37°C in an atmosphere of 5% CO_2_. After starved overnight in a serum‐free medium, cells were treated with Ang II (Millipore). FoxM1 inhibitor Siomycin A (Sigma‐Aldrich) was added to cells at 0.1‐1 μmol/L for 0.5 hour prior to Ang II.

### 2.3 Western blot

Kidney tissue or cells were lysed in lysis buffer containing 2% SDS following centrifugation. The supernatant was separated on SDS‐polyacrylamide gels. Then, proteins were electrophoretically transferred to polyvinylidene fluoride (PVDF) membrane (Millipore) and blocked with 5% non‐fat milk. Proteins were detected with primary antibody against E‐cadherin (610181; BD Transduction Laboratories), Snail (3879; Cell Signaling Technology), Fibronectin (F3648; Sigma‐Aldrich), FoxM1 (ab1807.10; Abcam), collagen I (Col I) (ab34710; Abcam), Wnt1 (ab15251; Abcam), Wnt2b (ab15251; Abcam), Wnt3 (ab172612; Abcam) from Abcam (Cambridge, MA, USA), active β‐catenin (05‐665; Millipore), β‐catenin (51067‐2‐AP; Proteintech), Col I (14695‐1‐AP; Proteintech), α‐smooth muscle actin (α‐SMA) (14395‐1‐lg; Proteintech), GAPDH (60004‐1‐lg, Proteintech) from Proteintech. The bands were visualized by an ECL detection kit (AmerSham Biosciences) and quantitated via densitometric analysis using the Image J software (NIH).

### 2.4 RNA extraction and real‐time quantitative PCR

Total RNA was extracted using the TRIzol reagent (Life Technologies). For mRNA quantification, cDNA was synthesized using PrimeScript™ RT reagent Kit (Takara) and quantitative PCR was performed on Applied Biosystems 7300 Plus using SYBR Green PCR master mix (Toyobo). The primers sequences were listed in Supplemental Table S2. The relative abundance of target mRNA was normalized to the glyceraldehyde‐3‐phosphate dehydrogenase (GAPDH).

### 2.5 Renal histology and Immunohistochemistry staining

Mouse kidneys fixed in 10% formalin were embedded into paraffin and cut into 4‐μm thick sections. HE and Masson's trichrome staining were conducted following a protocol described previously. The tubular injury was assessed by HE staining based on tubular changes including tubule dilation, epithelial swelling, loss of brush borders and protein cast formation.^19^ Masson's trichrome staining was used to evaluate interstitial fibrosis. Fibrotic areas in 5 discretionary fields of each mouse were quantified. The average percentage of fibrotic area relative to the total area was regarded as fibrotic score. The mouse kidneys and human kidney biopsy sections were stained using specific anti‐FoxM1 antibody (ab1807.10; Abcam).

### 2.6 Construction of Vectors

Rat FoxM1 gene was cloned into pcDNA3.1 as described previously.^10^ Wnt1, Wnt2b and Wnt3 promoter fragments were amplified from rat genomic DNA with appropriate primers. Mutated Wnt1, Wnt2b and Wnt3 promoter regions were constructed by deleting FoxM1 binding site on the corresponding promoter region. The PCR products were cloned into the luciferase vector pGL3‐basic. Adenoviral expression vector GV314 encoding rat FoxM1 gene (Ad‐3 × FLAG‐EGFP‐FoxM1) was constructed according to the manufacturer's protocol. Transduction was confirmed via FoxM1 expression. All constructs were verified by sequencing.

### 2.7 RNA interference

FoxM1 siRNA (5′‐GAAUCAAGAUUAUCAACCA‐3′), β‐catenin siRNA (5′‐GCAACUCAAGCAGAUCUAA‐3′) were designed and synthesized by Biotend (Shanghai, China). NRK‐52E cells were transfected with FoxM1, β‐catenin siRNA (50 nM) or non‐silencing negative siRNA (50 nM) using Lipofectamine RNAiMAX reagent (13778030; Thermo Fisher Scientific) according to the manufacturer's instructions.

### 2.8 Immunofluorescence staining

Double immunofluorescence staining was implemented as established protocols.^10^ The mouse kidney sections were incubated with anti‐FoxM1 (ab1807.10; Abcam) and anti‐AQP1 (ab9566; Abcam). NRK‐52E cells were incubated with adenovirus expressing rat FoxM1 gene (Ad‐FoxM1) or empty vector (Ad‐null), then followed by fixation at 37°C using 4% paraformaldehyde for 20 minutes. Fixed cells were hybridized with primary antibodies against FoxM1 (sc‐271746; Santa Cruz) and active β‐catenin (05‐665; Millipore) at 4°C overnight. Then, secondary antibodies (Proteintech) and 4′,6‐Diamidino‐2‐phenylindole (DAPI; Beyotime) visualized primary antibodies and nuclei, respectively. Cells were viewed using a confocal biological microscope (ZEISS).

### 2.9 Isolation of nuclear proteins

Cells were pre‐treated with and without 1 μmol/L of Siomycin A for 0.5 hour prior to Ang II. Then, cells were harvested and conducted nuclear protein extraction using the Nuclear and Cytoplasmic Protein Extraction Kit (P0028; Beyotime) based on the manufacturer's instructions. The nuclear fraction was dissociated using nuclear lysis buffer and subjected to Western blot.

### 2.10 Luciferase assay

The luciferase assay was performed as described previously. Briefly, human embryonic kidney (HEK) 293T cells were co‐transfected of FoxM1 overexpression plasmid and β‐galactosidase (β‐gal) plasmid with the wild‐type Wnt1, Wnt2b, Wnt3 or mutant Wnt1, Wnt2b and Wnt3 luciferase reporter plasmid using Lipofectamine 3000 reagent. After cultured for 48 hours, the cells were harvested, and luciferase activity was measured with the Luciferase Assay Kit (Promega). Relative luciferase activity was calculated as the ratio of Luc/β‐gal activity.

### 2.11 Chromatin immunoprecipitation assay

The assay was performed with EZ‐Chromatin immunoprecipitation (ChIP)TM assay kit (17‐371; Millipore) according to the manufacturer's protocols. In brief, NRK‐52E cells were transfected with adenovirus harbouring FoxM1 for 48 hours. The crosslink was performed by adding 1% formaldehyde to the lysate for 10 minutes at room temperature and terminated by glycine. After sonicated, the DNA fragments in cell lysates were subjected to immunoprecipitation using rabbit anti‐FLAG antibody (14793; Cell Signaling Technology) or normal rabbit IgG (as a control). The reverse crosslinked complexes were purified and amplified by real‐time qPCR. PCR primers to analyse Wnt1, Wnt2b and Wnt3 were listed in Supplemental Table S3. Fold enrichment of bound area was defined by DNA amount relative to the total DNA.

### 2.12 Human kidney tissue samples

Human residual kidney biopsy samples were obtained from the Department of Nephrology, Tongji Hospital, Tongji University, Shanghai, China. Samples included different types of CKD with renal fibrosis (membranous nephropathy, MN; IgA nephropathy, IgAN; diabetic nephropathy, DN,) while minimal change disease (MCD) was used as control. All operations were conformed to the guidelines established by the Science Council of China and approved by the Medical Ethics Committee of Tongji Hospital Ethics Committee K‐W‐2019‐008.

### 2.13 Statistical analysis

Data are shown as the mean ± SEM. The analysis of data was performed by SPSS 19.0 (Chicago, IL, USA). Comparisons between groups were performed by one‐way ANOVA with post hoc analysis using Tukey's test. *P* < .05 was considered as statistically significant.

## 3 RESULTS

### 3.1 FoxM1 was increased in UUO or FA‐induced fibrotic kidneys

**Figure 1:**
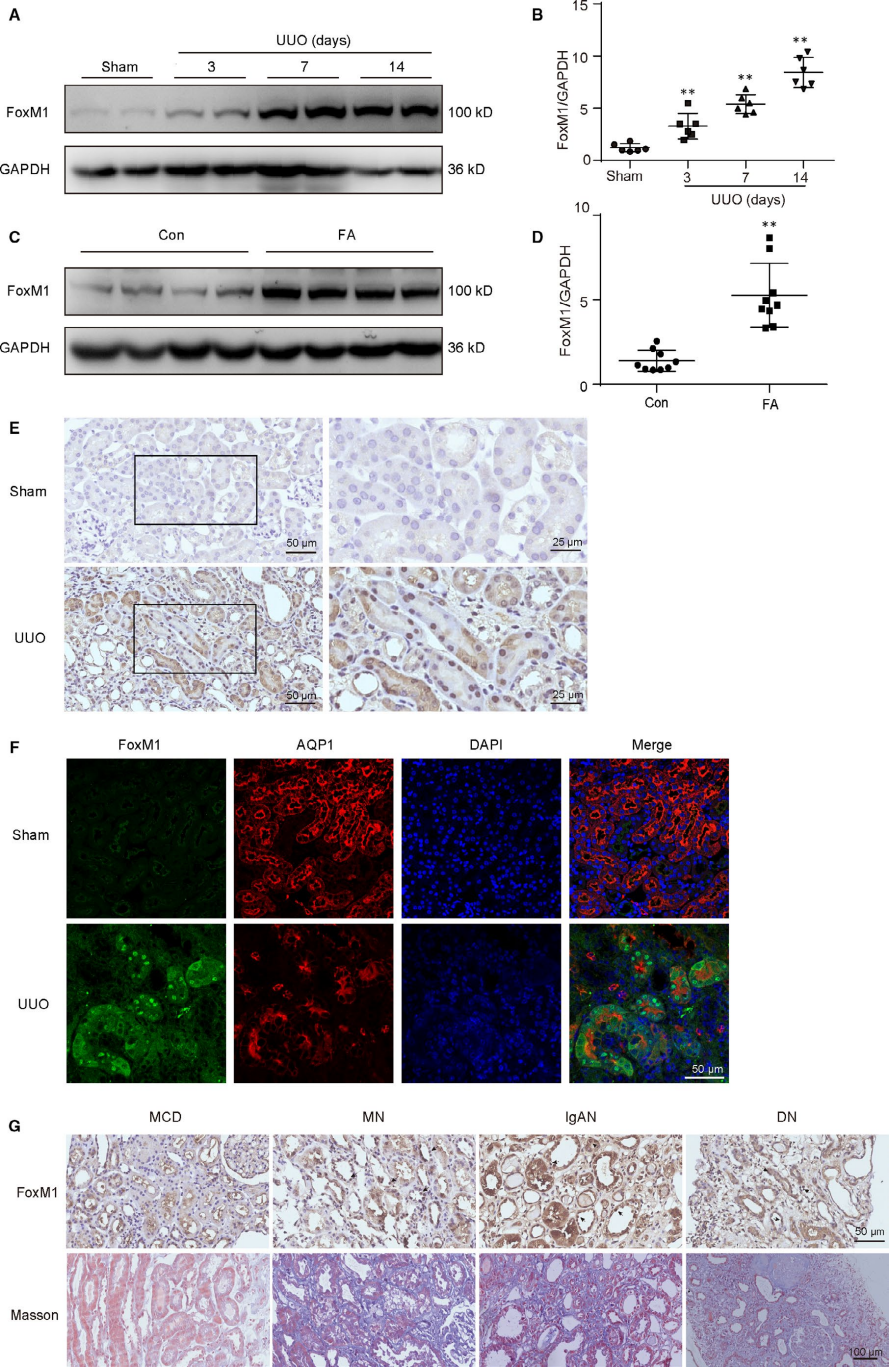
FoxM1 was elevated in tubular epithelial cells from UUO or FA‐induced fibrotic kidneys. A, Representative Western blot of FoxM1 in UUO mice kidneys. B, Quantitative data for FoxM1 in mice kidneys at 3, 7 and 14 d after UUO (n = 6). **P < .01 vs Sham. C, Representative Western blot of FoxM1 in FA mice kidneys. D, Quantitative data for FoxM1 in mice kidneys at 28 d after FA treatment (n = 9). **P < .01 vs Con. E, Immunohistological staining of FoxM1 in sham and UUO mice at 14 d after surgery. Scale Bar: 50 μm (left panels) and 25 μm (right panels). F, Co‐immunofluorescence staining of FoxM1 (green) with AQP1 (red) and DAPI (blue). Scale bar: 50 μm. G, Immunohistological staining of FoxM1 (Scale bar: 50 μm) and Masson staining (Scale bar: 100 μm) in kidney biopsies from CKD patients. AQP1, aquaporin‐1; CKD, chronic kidney disease; Con, control; DAPI, 4′6‐diamidino‐2‐phenylindole; DN, diabetic nephropathy; FA, folic acid; IgAN, IgA nephropathy; MCD, minimal change disease; MN, membranous nephropathy; UUO, unilateral ureteral obstruction

The expression of transcriptional factor FoxM1 in fibrotic mice was detected by Western blot. The result showed that FoxM1 was significantly increased at 3 days after UUO surgery and sustained to the end of experiment (14 days) (Figure 1A,B). The elevation of FoxM1 was also observed in FA‐induced renal fibrosis animals (Figure 1C,D). Immunohistochemistry result showed that FoxM1 was mainly distributed in renal tubules (Figure 1E). To identify the tubular segment specificity of FoxM1 expression in the kidneys, we performed double‐immunostaining for FoxM1 (green) and aquaporin‐1 (AQP1, red), a marker of proximal tubule. The result indicated that the increased FoxM1 was predominantly localized in nucleus of proximal renal tubules (Figure 1F).

To identify the relevance of FoxM1 in clinic, the expression of FoxM1 was observed in residual kidney biopsies from CKDs patients with renal fibrosis. Compared with MCD, FoxM1 was remarkably increased in biopsy samples of MN, IgAN and DN patients with renal fibrosis. Immunohistochemistry result showed that the expression of FoxM1 was also principally located in the nucleus of tubular epithelial cells (Figure 1G).

### 3.2 Selective FoxM1 inhibitor Siomycin A significantly alleviated fibrosis in UUO mice

**Figure 2:**
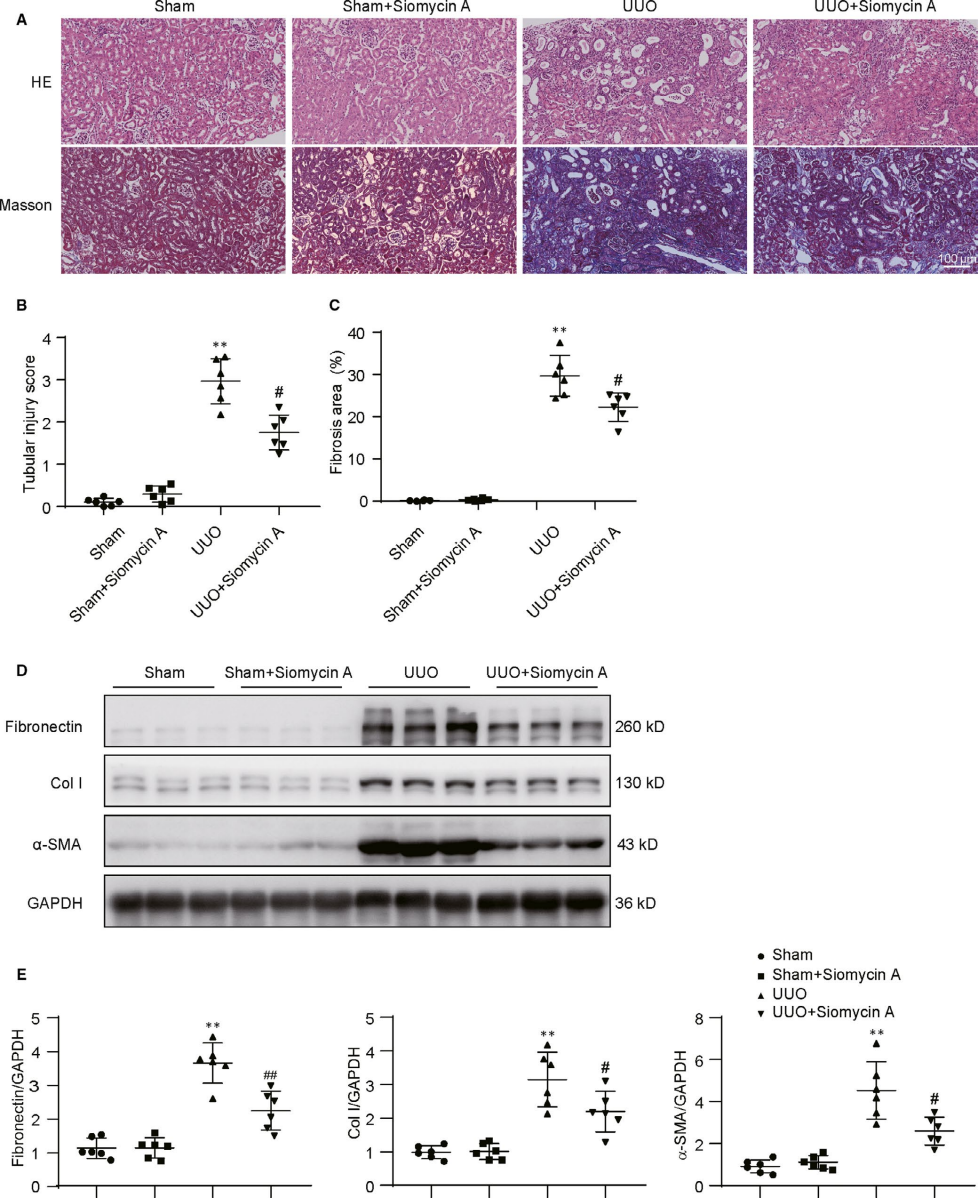
The FoxM1 inhibitor Siomycin A attenuated renal fibrosis in UUO mice. A, Representative HE and Masson stained kidney images . Scale bar: 100 μm. B, Quantitative determination of tubular injury score (n = 6). **P < .01 vs Sham. ^#^ *P* < .05 vs UUO. C, Assessment of interstitial fibrosis based on Masson staining (n = 6). **P < .01 vs Sham. ^#^ *P* < .05 vs UUO. D, Representative Western blot E, Quantitative data of fibronectin, Col I and α‐SMA from different groups (n = 6). **P < .01 vs Sham. ^#^ *P* < .05, ^##^ *P* < .01 vs UUO. Col I, collagen I; HE, hematoxylin‐eosin; Masson, Masson's trichrome; UUO, unilateral ureteral obstruction; α‐SMA, α‐smooth muscle actin

To identify the effect of FoxM1 on renal fibrosis, Siomycin A, a selective FoxM1 inhibitor, was injected intraperitoneally into UUO mice. HE and Masson staining results showed that the injection of Siomycin A did not exhibit obvious effect in sham mice. The obstructed kidneys displayed typical fibrosis characteristics, which was evidenced as the loss of brush border, tubule dilatation and cast formation by HE staining analysis and interstitial extracellular matrix deposition by Masson staining analysis. Inhibition of FoxM1 by Siomycin A delivery significantly alleviated the pathological changes in obstructed kidneys (Figure 2A‐C). Western blot result revealed that the expression of extracellular matrix components, fibronectin, Col I and renal fibrosis maker α‐SMA was significantly increased in the obstructed kidneys. Siomycin A treatment did not obviously affect the expression of extracellular matrix components in sham mice, but effectively inhibited the increase in fibronectin, Col I and α‐SMA in obstructed kidneys (Figure 2D,E).

### 3.3 Overexpression of FoxM1‐induced mRNA and protein expression of Wnts in renal tubular epithelial cells

**Figure 3:**
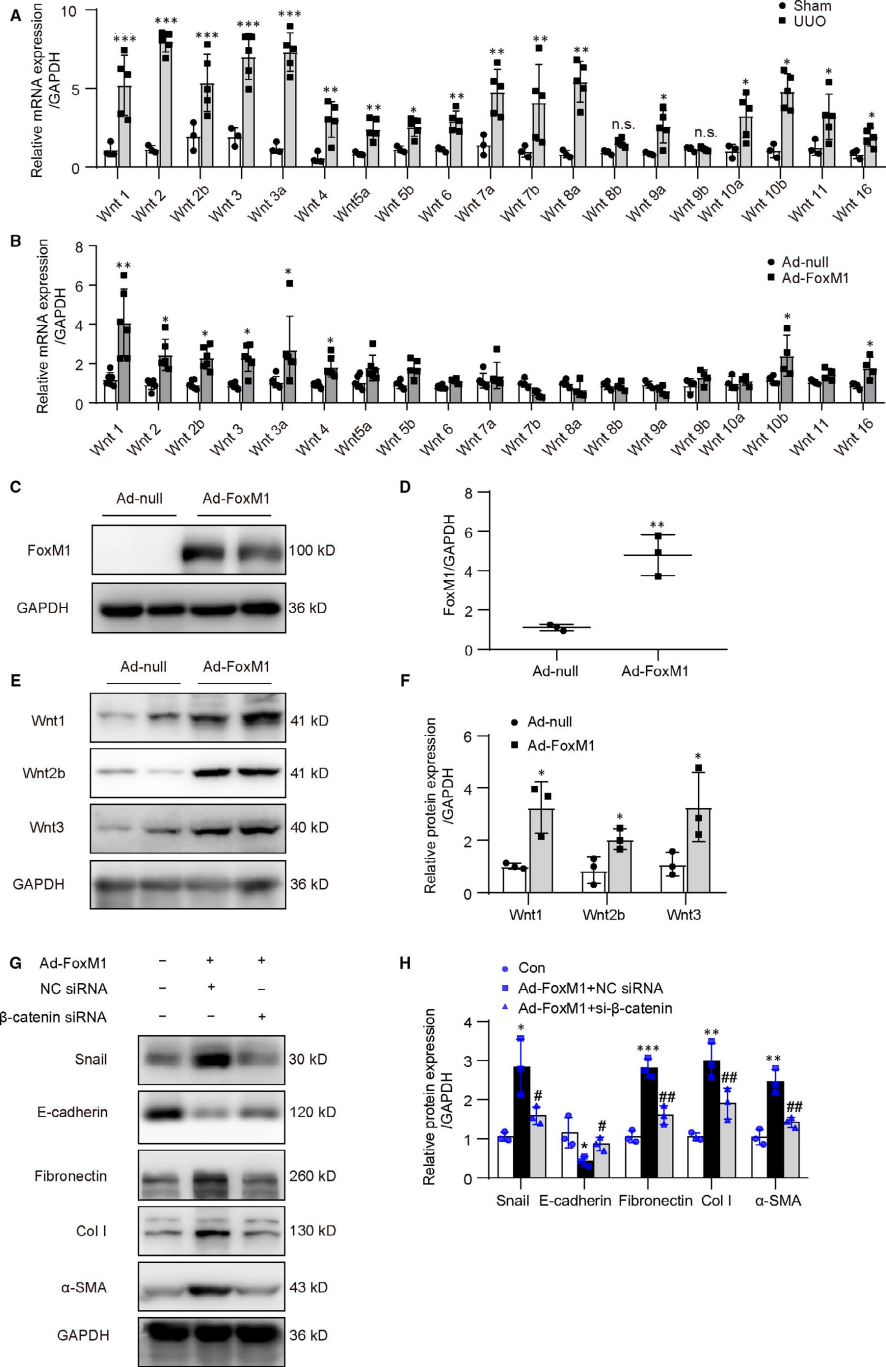
Overexpression of FoxM1 increased the expression of Wnts in tubular epithelial cells. A, qPCR analysis for Wnts mRNA in the kidneys at 14 d after UUO (n = 3‐5 mice per group). *P < .05, **P < .01, ***P < .001 vs Sham. B, qPCR analysis for Wnts mRNA in NRK‐52E after transfection of Ad‐FoxM1 or Ad‐null for 48 h (n = 4‐6). *P < .05, **P < .01 vs Ad‐null. C and E, Representative Western blot of FoxM1 and Wnts in NRK‐52E. D and F, Quantitative data from C and E, respectively, (n = 3). *P < .05, **P < .01 vs Ad‐null. G, Cells were transfected with Ad‐FoxM1, NC siRNA or β‐catenin siRNA. Representative Western blot of snail, E‐cadherin and extracellular proteins. H, Quantitative data from G (n = 3). *P < .05, **P < .01, ***P < .001 vs Con. ^#^ *P* < .05, ^##^ *P* < .01 vs Ad‐FoxM1 + NC siRNA. Ad‐FoxM1, recombinant adenovirus containing rat FoxM1 gene; Ad‐null, empty adenovirus vectors; Col I, collagen I; Con, control; UUO, unilateral ureteral obstruction; α‐SMA, α‐smooth muscle actin

The changes of Wnts in the obstructed kidneys were detected by qPCR. The result showed that 17 of 19 Wnts (except Wnt 8b and Wnt 9b) were significantly up‐regulated in the obstructed kidneys at 14 days after surgery (Figure 3A).

Bioinformatic analysis result showed that there are computative binding sites of FoxM1 on all human Wnts and 13 of 19 rat Wnts promoter regions. (Supplemental Table S1). To identify whether FoxM1 regulated the expression of Wnts, an adenovirus vector harbouring FoxM1 gene (Ad‐FoxM1) was transfected into cultured NRK‐52E. qPCR result showed that 8 of 19 Wnts mRNA were apparently increased following Ad‐FoxM1 transfection (Figure 3B). Among the increased Wnts, the changes in Wnt1, Wnt2b and Wnt3 were further validated by Western blot. After FoxM1 overexpression, FoxM1 was significantly increased (Figure 3C,D). Consistent with the changes in mRNA, Wnt1, Wnt2b and Wnt3 protein levels were dramatically increased (Figure 3E,F). We also identified the changes in major ECM proteins and fibrosis‐related gene expressions after FoxM1 overexpression. The result showed that transfection of Ad‐FoxM1 significantly increased the expression of fibronectin, Col I, α‐SMA and snail, by contrast, decreased the expression of E‐cadherin. Knock‐down on β‐catenin by siRNA reversed the changes induced by FoxM1 overexpression (Figure 3G,H). These data indicated that the overexpression of FoxM1 promoted multi‐Wnts and β‐catenin‐mediated fibrotic gene expressions in renal tubular epithelial cells.

### 3.4 Knock‐down on FoxM1 suppressed Ang II‐induced the expression of Wnts and fibrosis‐related gene expressions in vitro

**Figure 4:**
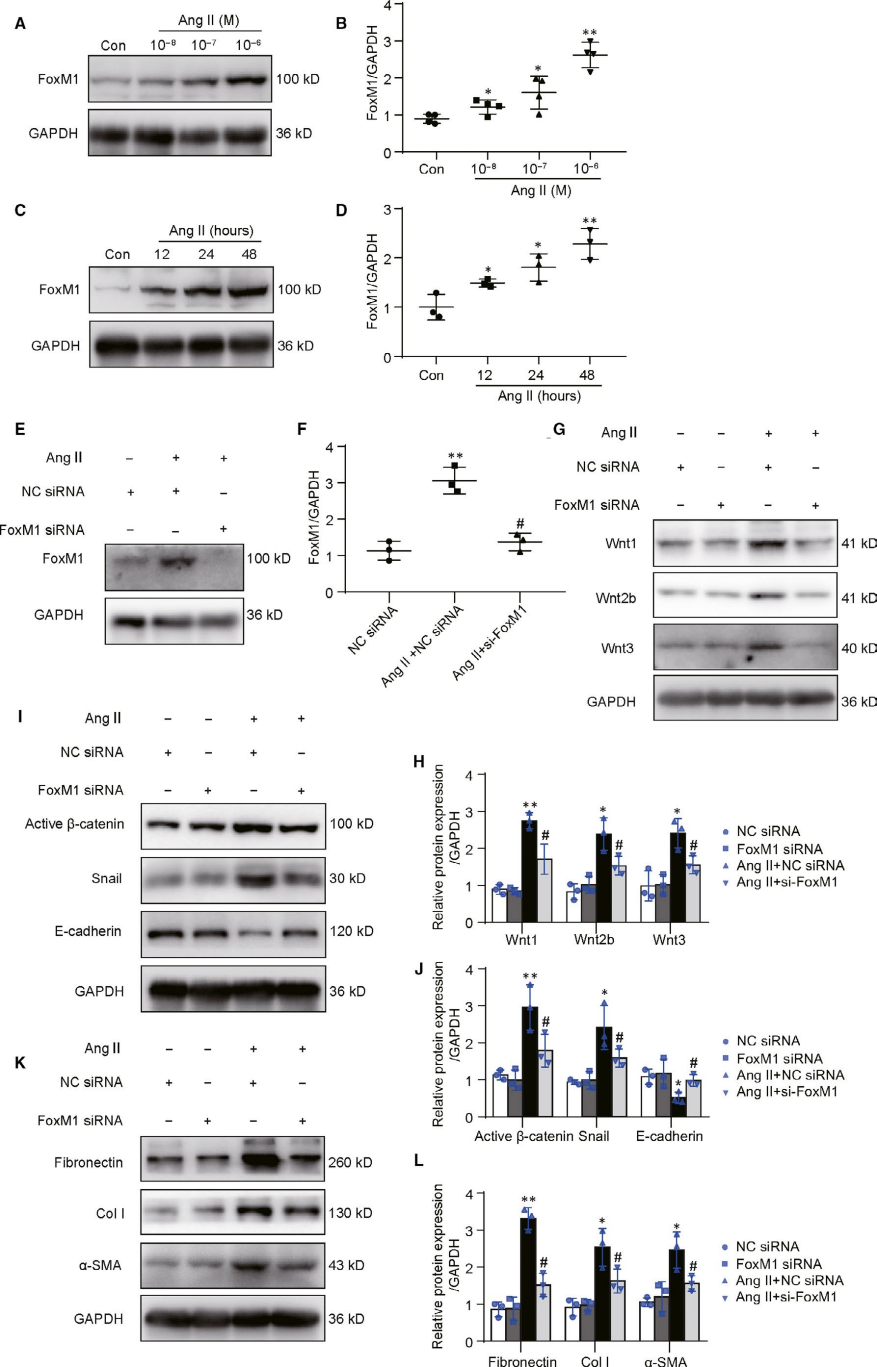
Knock‐down on FoxM1 reduced Ang II‐induced Wnts expression and β‐catenin pathway activation in NRK‐52E. A, Representative Western blot of FoxM1. Cells were treated with Ang II at a concentration of 10^−8^, 10^−7^, 10^−6^ M for 48 h. B, Quantitative data for FoxM1 after Ang II treatment (n = 4). *P < .05, **P < .01 vs Con. C, Representative Western blot of FoxM1. Cells were treated with Ang II (10^−6^ M) and harvested at 12, 24 and 48 h. D, Quantitative data for FoxM1 after Ang II treatment (n = 3). *P < .05, **P < .01 vs Con. E, G, I and K, Representative Western blot of FoxM1, Wnts, β‐catenin activation and fibrosis‐associated genes. Cells were pre‐treated with FoxM1 siRNA (50 nmol/L) or NC siRNA (50 nmol/L) for 24 h and then exposed to Ang II (10^−6^ M) for 24 h. F, H, J and L, Quantitative data from E, G, I and K, respectively (n = 3). *P < .05, **P < .01 vs NC siRNA. ^#^ *P* < .05 vs Ang II + NC siRNA. Con, control; Col I, collagen I; α‐SMA, α‐smooth muscle actin

To verify the result observed in vivo, cultured renal tubular epithelial cells were treated with Ang II, the major effector in renin‐angiotensin system.^20^ As showed in Figure 4A‐D, Ang II‐induced significant increase in FoxM1 expression in a dose and time‐dependent manner. Western blot result showed that FoxM1 siRNA markedly reversed the increase of FoxM1 induced by Ang II (Figure 4E,F). Meanwhile, the increases of Wnt1, Wnt2b and Wnt3 were attenuated by knock‐down on FoxM1 (Figure 4G,H). Ang II‐induced increase in active β‐catenin, snail, fibronectin, Col I, α‐SMA and decrease in E‐cadherin were also reversed by knock‐down on FoxM1 (Figure 4I‐L).

### 3.5 FoxM1 inhibitor Siomycin A suppressed Ang II‐induced Wnts and fibrosis‐related gene expressions in vitro

**Figure 5:**
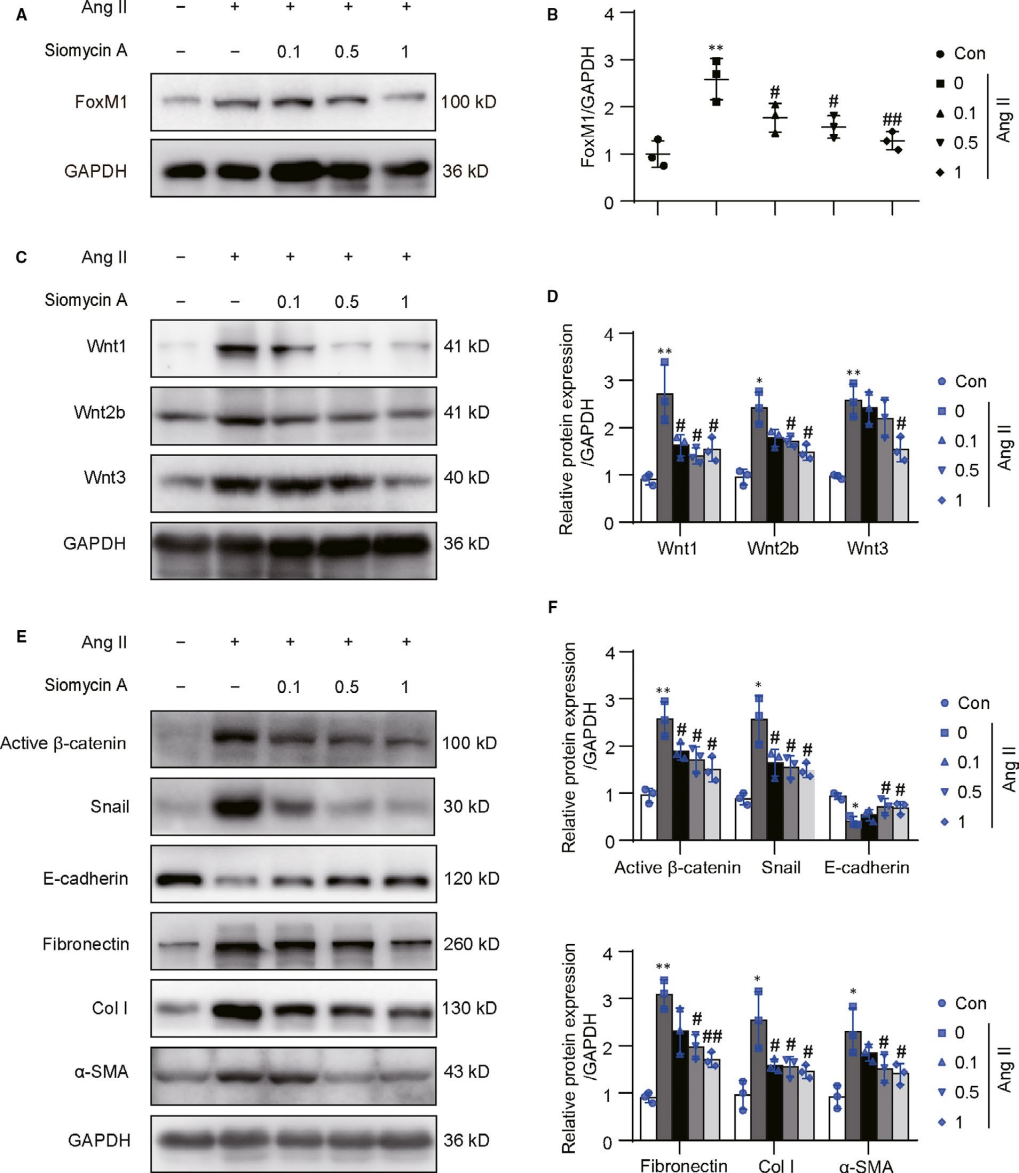
Siomycin A diminished Ang II‐induced β‐catenin activation and profibrotic responses in NRK‐52E. Cells were pre‐treated with Siomycin A (0, 0.1, 0.5, 1 μM) for 0.5 h and then treated with Ang II (10^−6^ M) for 48 h. A, C and E, Representative Western blot, B, quantitative data for FoxM1, D, quantitative data for Wnt1, Wnt2b, Wnt3 and F, quantitative data for active β‐catenin, Snail, E‐cadherin, fibronectin, Col I and α‐SMA (n = 3). *P < .05, **P < .01 vs Con. ^#^ *P* < .05, ^##^ *P* < .01 vs Ang II. Con, control; Col I, collagen I; α‐SMA, α‐smooth muscle actin

Consistent with the results from FoxM1 knock‐down, pharmacological inhibition of FoxM1 by Siomycin A alleviated Ang II‐induced up‐regulation of FoxM1, Wnt1, Wnt2b and Wnt3 in a dose‐dependent manner (Figure 5A‐D). Meanwhile, Ang II‐induced changes, including the increases in active β‐catenin, snail, fibronectin, Col I, α‐SMA and decrease in E‐cadherin, were also inhibited in dose‐dependent manner (Figure 5E,F). These results suggested FoxM1‐mediated Ang II‐induced β‐catenin activation in renal epithelial cells.

### 3.6 FoxM1 augmented β‐catenin nuclear distribution via enhancing Wnts transcription in renal tubular epithelial cells

**Figure 6:**
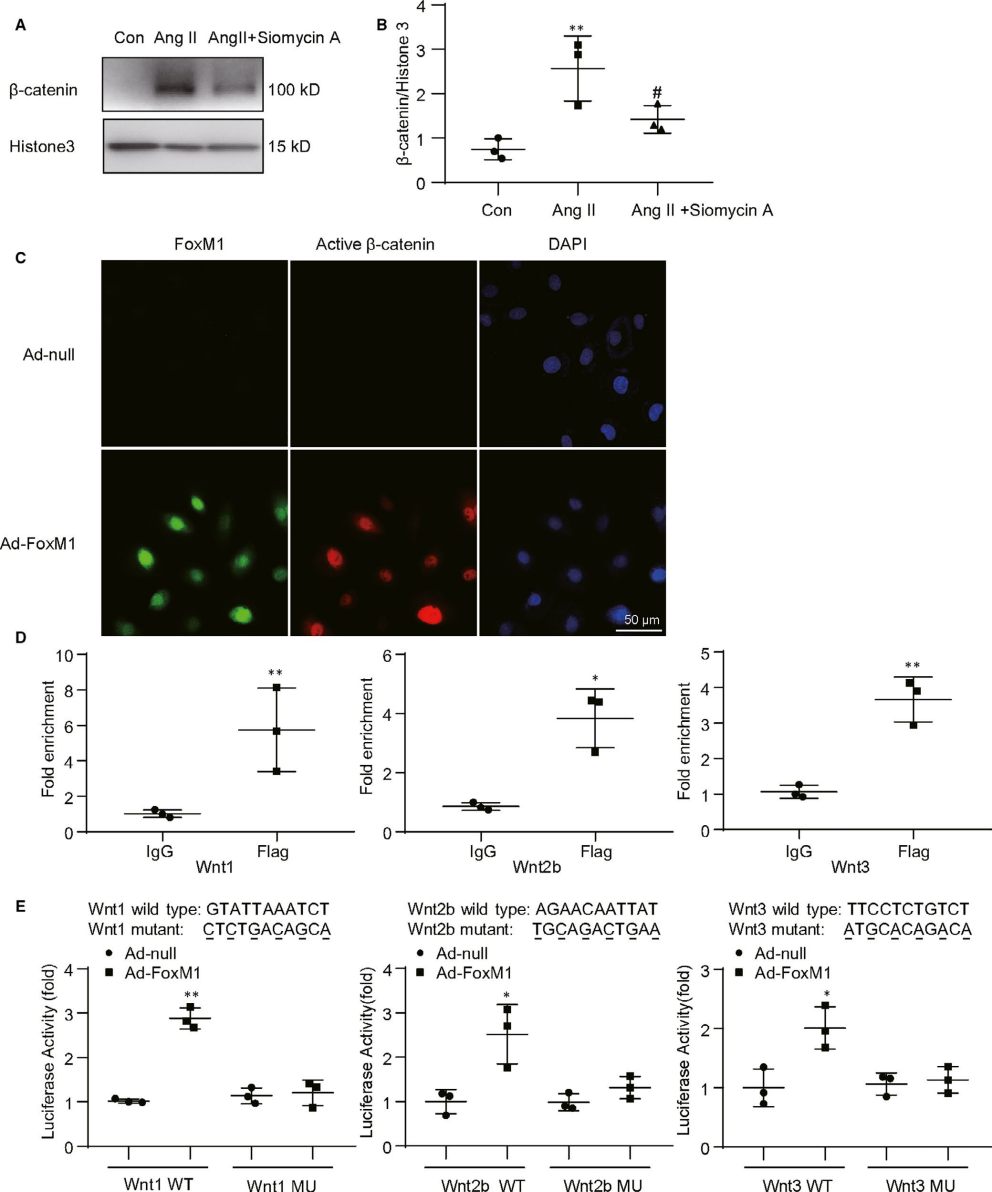
FoxM1 augmented nuclear distribution of β‐catenin and directly regulated Wnts transcription in renal tubular epithelial cells. A, Representative Western blot of β‐catenin in nuclear fractions. NRK‐52E were treated with Ang II (10^−6^ M) for 48 h with or without FoxM1 inhibitor Siomycin A. B, Quantitative data for β‐catenin in nuclear fractions (n = 3). **P < .01 vs Con. ^#^ *P* < .05 vs Ang II. C, Representative immunofluorescence images of FoxM1 (green), active β‐catenin (red) and DAPI (blue). Cells were infected with Ad‐FoxM1 or Ad‐null for 48 h. Bar = 50 μm. D, Chromatin immunoprecipitation assay was conducted with antibody against IgG or Flag and normalized to the input DNA. Amplifications of Wnt1, Wnt2b and Wnt3 were performed by qPCR (n = 3). *P < .05, **P < .01 vs IgG. E, HEK 293T cells were co‐transfected with the plasmids expressing FoxM1, β‐gal and luciferase promoter containing Wnt1, Wnt2b, Wnt3 WT or Wnt1, Wnt2b and Wnt3 MU promoter sequence. Luciferase activity was detected 48 h after transfection (n = 3). **P < .01 vs Wnt1 WT, *P < .05 vs Wnt2b WT or Wnt3 WT. Ad‐FoxM1, recombinant adenovirus containing rat FoxM1 gene; Ad‐null, empty adenovirus vectors; Con, control; DAPI, 4′, 6‐diamidino‐2‐phenylindole; MU, luciferase promoter containing mutant promoter sequences of Wnts; WT, luciferase promoter containing wild‐type promoter sequences of Wnts; β‐gal, β‐galactosidase

To identify the role of FoxM1 in β‐catenin activation, the nuclear β‐catenin was detected by Western blot. Exposure of the renal tubular epithelial cells to Ang II enhanced β‐catenin nuclear distribution, whereas pre‐treatment with Siomycin A evidently blunted β‐catenin nuclear translocation (Figure 6A,B). Overexpression of FoxM1 also induced a significant increase in β‐catenin nuclear distribution (Figure 6C).

ChIP‐qPCR assay was performed to determine whether FoxM1 directly regulates Wnts gene transcription. Compared with IgG, the precipitated Wnt1, Wnt2b and Wnt3 promoter DNAs were dramatically increased (Figure 6D). To further confirm the binding of FoxM1, original or mutant Wnt1, Wnt2b and Wnt3 promoter luciferase plasmids were co‐transferred into HEK 293T cells with FoxM1 overexpression plasmid or empty vector for 48 hours. When wide‐type (WT) Wnt1, Wnt2b or Wnt3 promoter luciferase plasmid was co‐transfected with FoxM1 overexpression vector, luciferase activity was significantly increased while the mutation of Wnt1, Wnt2b or Wnt3 promoter region by deleting FoxM1 binding site abolished the effect (Figure 6E). The data indicated that FoxM1 was able to bind to the promoter region of Wnt1, Wnt2b and Wnt3 and positively regulated the expression of those Wnts.

### 3.7 FoxM1 inhibition alleviated the Wnts and fibrotic‐related gene expressions in UUO mice

**Figure 7:**
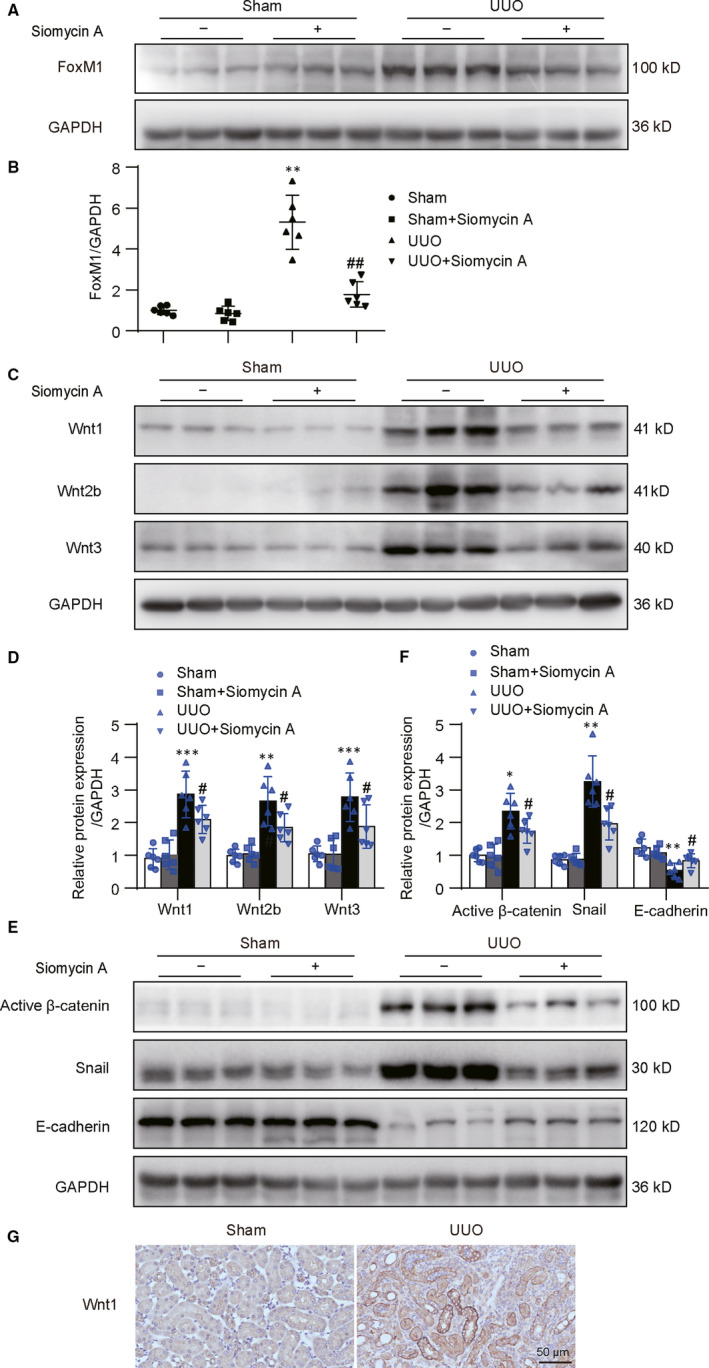
Siomycin A inhibited Wnt/β‐catenin pathway activation and fibrotic‐related gene expressions in UUO mice. A and C, Representative Western blot B and D, Quantitative analysis of renal FoxM1, Wnt1, Wnt2b and Wnt3 expression at 14 d following UUO (n = 6). **P < .01, ***P < .001 vs Sham. ^#^ *P* < .05, ^##^ *P* < .01 vs UUO. E, Representative Western blot F, Quantitative analysis of renal active β‐catenin, Snail and E‐cadherin expression at 14 d following UUO (n = 6). *P < .05, **P < .01 vs Sham. ^#^ *P* < .05 vs UUO. G, Representative immunohistological staining for Wnt1 in sham and UUO mice. Scale bar: 50 μm. UUO, unilateral ureteral obstruction

To observe the effects of FoxM1 on expressions of Wnts and renal fibrosis‐related genes, UUO mice were treated with FoxM1 inhibitor Siomycin A. The result showed that Siomycin A did not exhibit obvious effects on the expression of FoxM1 in sham‐operated kidneys, but dramatically abrogated the increase in FoxM1 in obstructed kidneys (Figure 7A,B). As expected, Siomycin A significantly attenuated the increase in Wnt1, Wnt2b and Wnt3 in UUO mice (Figure 7C,D). Similarly, Siomycin A administration did not exhibit obvious influence on β‐catenin activation, snail and E‐cadherin in sham‐operated kidneys, but significantly suppressed the increase in β‐catenin activation, snail and the decrease in E‐cadherin in UUO kidneys (Figure 7E,F). Besides, immunostaining confirmed the Wnt1 expression was increased in UUO mice and mainly distributed in the renal tubules (Figure 7G). These data supported the idea that FoxM1 promoted renal fibrosis via Wnts secretion and subsequently initiated β‐catenin pathway activation.

## 4 DISCUSSION

Under pathological conditions, original injury in renal tubular epithelial cells is a pivotal driver of secondary renal injury, including the development and progression of renal fibrosis. The injured epithelial cells switch into secretory phenotype and release plentiful fibrogenic and proinflammatory factors and thus promote renal fibrosis.^21^

It has been well documented that the activation of Wnt/β‐catenin plays a pivotal role in promoting renal fibrosis. As described in previous study, pharmacological inhibition of β‐catenin in tubular epithelium suppressed the progression of renal fibrosis under pathological circumstances.^5^, ^6^ Activation of β‐catenin requires molecular Wnts binding to their receptors.^9^ Conditional expression of Wnt1 in the proximal tubules effectively activated fibroblasts and promoted renal fibrosis.^22^ In human proximal tubular cells, overexpression of Wnt1 apparently activated β‐catenin nuclear translocation and promoted cellular senescence.^23^, ^24^ A latest study illustrated tubule‐specific deficiency of Wnts sufficiently reduced renal fibrosis.^25^, ^26^ All these studies indicate the renal tubular epithelial cells are an important source of Wnts in kidneys.

Nineteen Wnt family members have been identified. In the present study, our result showed that almost all Wnts expressions were increased in UUO‐induced fibrotic kidneys, which was consistent with the previous observations.^10^ This result also suggested subduction on any Wnt production and secretion may not be able to effectively block the progression of renal fibrosis. Thus, finding out the master switch on Wnt/β‐catenin signalling pathway becomes essential in manipulating renal fibrosis.

Here, we identified that FoxM1 was a key transcriptional regulator of multi‐Wnts. The expression of FoxM1 was increased in both UUO and FA‐induced fibrotic kidneys. The distribution of FoxM1 was mainly in renal tubular epithelial cells, the major source of Wnts. Pharmacological inhibition of FoxM1 expression significantly attenuated the progression of renal fibrosis. This result was accompanied by suppressing Wnt1, Wnt2b, Wnt3 elevation in obstructed kidneys, and alleviating phenotype changes in epithelial cells. In cultured epithelial cells, overexpression of FoxM1 up‐regulated 8 of 19 Wnts expressions, while knock‐down or inhibition on FoxM1 suppressed Ang II‐induced Wnts expression and β‐catenin nuclear translocation. ChIP‐qPCR and promoter luciferase activity assay results also suggested that multi‐Wnts might be downstream target genes of FoxM1. All these results prompt the idea that transcriptional factor FoxM1 is an important regulator for Wnts expressions. The increase in FoxM1 under pathological condition is implicated in renal fibrosis via up‐regulating multi‐Wnts expression in epithelial cells, then promoting the activation of β‐catenin signalling pathway. The preliminary observation on biopsies from patients with renal fibrosis suggested that the expression of FoxM1 was increased in different renal fibrosis and mainly located in renal tubular epithelial cells, which strengthened the clinical relevance of the study.

However, the increase in Wnts expressions induced by overexpression of FoxM1 in cultured renal tubular epithelial cells were not completely match what observed in UUO kidneys, which suggested that other factors other than FoxM1 might be involved in regulating Wnts expression. Moreover, FoxM1 was able to regulate β‐catenin transcription^27^, ^28^ and facilitate β‐catenin nuclear translocation.^29^, ^30^, ^31^ These studies indicated that the effect of FoxM1 in promoting renal fibrosis may not completely rely on regulating Wnts expressions. The results in the study suggest that FoxM1 is a pivotal regulator in promoting renal fibrosis, but what is line on the upstream of FoxM1 still need to be elucidated.

In conclusion, our data demonstrated that up‐regulated transcriptional factor FoxM1 stimulates multi‐Wnts transcription and then promotes renal fibrosis via activating Wnt/β‐catenin signalling pathway. FoxM1 might be a master switch to turn on the abnormal activation of Wnt/β‐catenin signalling pathway under pathological conditions.

## CONFLICT OF INTEREST

All the authors declare no conflict of interest.

## AUTHOR CONTRIBUTIONS

**Hongyan Xie:** Resources (lead); Software (lead); Supervision (lead); Validation (lead); Visualization (lead); Writing‐original draft (lead); Writing‐review & editing (lead). **Naijun Miao:** Software (supporting). **Dan Xu:** Validation (supporting). **Zhuanli Zhou:** Validation (supporting). **Jiayun Ni:** Software (supporting). **Fan Yin:** Visualization (supporting). **Yan‐Zhe Wang:** Software (supporting). **Qian Cheng:** Supervision (supporting). **Panpan Chen:** Software (supporting). **Jingyao Li:** Software (supporting). **Peiqing Zheng:** Visualization (supporting). **Li Zhoua:** Supervision (supporting). **Jun Liu:** Supervision (supporting). **Wei Zhang:** Supervision (supporting). **Xiao‐Xia Wang:** Supervision (supporting). **Limin Lu:** Writing‐review & editing (lead).

## Supporting information

Table S1

Table S2

Table S3

## Data Availability

The data sets used in the current study are available from the first author and corresponding author on reasonable request.
