# Supplementary material for: FoxM1 promotes Wnt/β‐catenin pathway activation and renal fibrosis via transcriptionally regulating multi‐Wnts expressions
Source: J Cell Mol Med. 2021 Jan 12;25(4):1958–71. doi: 10.1111/jcmm.15948 (PMC7882937; doi:10.1111/jcmm.15948)
Supplement: Supplementary file 1 — Table S1 [file JCMM-25-1958-s001.docx]

Supplemental Table S1. FoxM1 potential binding sites on human and rat Wnt promotors

| FoxM1 potential binding sites on human Wnt promotors | | | |
| --- | --- | --- | --- |
| Human-promotor | Transcription factor | potential binding sites | Numbers |
| Wnt1 | FoxM1 | Yes | 6 |
| Wnt2 | FoxM1 | Yes | 5 |
| Wnt2b | FoxM1 | Yes | 3 |
| Wnt3 | FoxM1 | Yes | 16 |
| Wnt3a | FoxM1 | Yes | 9 |
| Wnt4 | FoxM1 | Yes | 13 |
| Wnt5a | FoxM1 | Yes | 9 |
| Wnt5b | FoxM1 | Yes | 17 |
| Wnt6 | FoxM1 | Yes | 4 |
| Wnt7a | FoxM1 | Yes | 9 |
| Wnt7b | FoxM1 | Yes | 16 |
| Wnt8a | FoxM1 | Yes | 15 |
| Wnt8b | FoxM1 | Yes | 23 |
| Wnt9a | FoxM1 | Yes | 14 |
| Wnt9b | FoxM1 | Yes | 16 |
| Wnt10a | FoxM1 | Yes | 4 |
| Wnt10b | FoxM1 | Yes | 5 |
| Wnt11 | FoxM1 | Yes | 1 |
| Wnt16 | FoxM1 | Yes | 12 |

| FoxM1 potential binding sites on rat Wnt promotors | | | |
| --- | --- | --- | --- |
| Rat-promotor | Transcription factor | potential binding sites | Numbers |
| Wnt1 | FoxM1 | Yes | 1 |
| Wnt2 | FoxM1 | Yes | 1 |
| Wnt2b | FoxM1 | Yes | 3 |
| Wnt3 | FoxM1 | Yes | 1 |
| Wnt3a | FoxM1 | Yes | 1 |
| Wnt4 | FoxM1 | Yes | 1 |
| Wnt5a | FoxM1 | No | 0 |
| Wnt5b | FoxM1 | No | 0 |
| Wnt6 | FoxM1 | Yes | 1 |
| Wnt7a | FoxM1 | Yes | 1 |
| Wnt7b | FoxM1 | Yes | 1 |
| Wnt8a | FoxM1 | Yes | 3 |
| Wnt8b | FoxM1 | Yes | 1 |
| Wnt9a | FoxM1 | No | 0 |
| Wnt9b | FoxM1 | No | 0 |
| Wnt10a | FoxM1 | Yes | 1 |
| Wnt10b | FoxM1 | No | 0 |
| Wnt11 | FoxM1 | No | 0 |
| Wnt16 | FoxM1 | Yes | 1 |
