# Supplementary material for: FoxM1 promotes Wnt/β‐catenin pathway activation and renal fibrosis via transcriptionally regulating multi‐Wnts expressions
Source: J Cell Mol Med. 2021 Jan 12;25(4):1958–71. doi: 10.1111/jcmm.15948 (PMC7882937; doi:10.1111/jcmm.15948)
Supplement: Supplementary file 2 — Table S2 [file JCMM-25-1958-s002.docx]

Supplemental Table S2. Primers used for quantitative Real-time PCR

| **Mouse**  **gene** | **Primer Sequence 5’ to 3’**  **Forward Reverse** | |
| --- | --- | --- |
| Wnt1 | GCCCTAGCTGCCAACAGTAGT | GAAGATGAACGCTGTTTCTCG |
| Wnt2 | AGAGTGCCAACACCAGTTCC | TACAGGAGCCACTCACACCA |
| Wnt2b | TTGTGTCAACGCTACCCAGA | ACCACTCCTGCTGACGAGAT |
| Wnt3 | GGGGCGTATTCAAGTAGCTG | GTAGGGACCTCCCATTGGAT |
| Wnt3a | TTCTTACTTGAGGGCGGAGA | CTGTCGGGTCAAGAGAGGAG |
| Wnt4 | CGAGGAGTGCCAATACCAGT | GTCACAGCCACACTTCTCCA |
| Wnt5a | CCCAGTCCGGACTACTGTGT | TTTGACATAGCAGCACCAGTG |
| Wnt5b | TCTCCGCCTCACAAAAGTCT | CACAGACACTCTCAAGCCCA |
| Wnt6 | TTCGGGGATGAGAAGTCAAG | CGGCACAGACAGTTCTCCTC |
| Wnt7a | GACAAATACAACGAGGCCGT | GGCTGTCTTATTGCAGGCTC |
| Wnt7b | ACAGGAGGGTGGGGATAGA | GAAACAGCCCAGGAAACCGT |
| Wnt8a | CTGACTACTGCAACCGCAAC | TGACAGTGCAACACCACTGA |
| Wnt8b | CCAGAGTTCCGGGAGGTAG | GAGATGGAGCGGAAGGTGT |
| Wnt9a | CCCCTGACTATCCTCCCTCT | GATGGCGTAGAGGAAAGCAG |
| Wnt9b | GGGTGTGTGTGGTGACAATC | TCCAACAGGTACGAACAGCA |
| Wnt10a | GCGCTCCTGTTCTTCCTACT | ATGCCCTGGATAGCAGAGG |
| Wnt10b | TCAGTCGGGCTCTAAGCAAT | TGGTGCTGACACTCGTGAAC |
| Wnt11 | TGCTTGACCTGGAGAGAGGT | AGCCCGTAGCTGAGGTTGT |
| Wnt16 | CCCTCTTTGGCTATGAGCTG | TACTGGACATCATCCGAGCA |
| GAPDH | AACTTTGGCATTGTGGAAGG | ACACATTGGGGGTAGGAACA |

| **Rat**  **gene** | **Primer Sequence 5’ to 3’**  **Forward Reverse** | |
| --- | --- | --- |
| Wnt1 | AGCGACGACTGATCCGACA | GAGGTGATTGCGAAGATAAACG |
| Wnt2 | GGCCTTTGTTTACGCCATCT | CTTGCCACTCCCTTTCTTCTTT |
| Wnt2b | GTCAACACCAGTTCCGTCATCA | ATAGACAAACGCTGCCTCCC |
| Wnt3 | AACTTTTGTGAGCCCAACCC | ACCAGTGGAAGACGCAATGAC |
| Wnt3a | CGGCAGTTGCGAAGTGAAG | TTGGGCTCGCAGAAGTTAGG |
| Wnt4 | CTCGTCTTCGCCGTGTTCTC | ACATCTGCACCTGCCTCTGG |
| Wnt5a | AACTGGCGGGACTTTCTCAA | CGGAACTGGTACTGGCACTCTT |
| Wnt5b | CCATGCGTATCACCCGTCA | GGTCTCATTTCGCAGGCAGTA |
| Wnt6 | ATAACAACGAGGCAGGCAGAC | CAGAGCACAGGAACCCGAAA |
| Wnt7a | AGCTAGGCTACGTGCTCAAGG | CGGTAGGACAGGGGCTTCTT |
| Wnt7b | CCGCTATGGCATTGACTTTTC | TGGTAGTACATGAGCCCGACAC |
| Wnt8a | CAGTCATGTACGCTGTCACCAA | GGCATCCTTTCCTTTCTCCAA |
| Wnt8b | GCAGTTTGTGGACGCCCTAG | GTTTCATGGTGCCCTTGACC |
| Wnt9a | GTGGAGGCTGTGAGCATGAGT | AGATGGCGTAGAGGAAAGCAGT |
| Wnt9b | CACCCATGTGGGCATCAAG | CAGCCGTGTCATAGCGTAGC |
| Wnt10a | ATGAGTGCCAGCATCAGTTCC | CGCAAGCCTTCAGTTTACCC |
| Wnt10b | TCAGTCGGGCTCTAAGCAAT | ACTCGTGAACGGCGATGTG |
| Wnt11 | GCTGACATGCGCTGGAACT | CATACACGAAGGCTGACTCCC |
| Wnt16 | AGAGGTGGAACTGTATGGTCGC | GTCTCCTTGGTGCCGCTACT |
| GAPDH | TGACAACTTTGGCATCGTGG | GGGCCATCCACAGTCTTCTG |
