# Supplementary material for: FoxM1 promotes Wnt/β‐catenin pathway activation and renal fibrosis via transcriptionally regulating multi‐Wnts expressions
Source: J Cell Mol Med. 2021 Jan 12;25(4):1958–71. doi: 10.1111/jcmm.15948 (PMC7882937; doi:10.1111/jcmm.15948)
Supplement: Supplementary file 3 — Table S3 [file JCMM-25-1958-s003.docx]

Supplemental Table S3. Primers used for chromatin immunoprecipitation assay

| **Rat**  **gene** | **Primer Sequence 5’ to 3’**  **Forward Reverse** | |
| --- | --- | --- |
| Wnt1 Promoter | CTGGGCAGGTACAGTGGCATA | AGAAGCTGGCTGGGTTTGG |
| Wnt2b Promoter | TTGAGGCTGGAAGTTTATTTTGAGA | GACTGCAAGAGCACTCAACCAC |
| Wnt3 Promoter | TTGCCTGCTGTTCCTTAGCC | GATAGAGACACTGAGACGGAAAAC |
